# Pathophysiological Role of Histamine H4 Receptor in Cancer: Therapeutic Implications

**DOI:** 10.3389/fphar.2019.00556

**Published:** 2019-06-05

**Authors:** Melisa B. Nicoud, Karina Formoso, Vanina A. Medina

**Affiliations:** ^1^Laboratory of Tumor Biology and Inflammation, Institute for Biomedical Research (BIOMED), School of Medical Sciences, Pontifical Catholic University of Argentina (UCA), and the National Scientific and Technical Research Council (CONICET), Buenos Aires, Argentina; ^2^Pharmacology and Function of Ionic Channels Laboratory, Institute for Biomedical Research (BIOMED), School of Medical Sciences, Pontifical Catholic University of Argentina (UCA), and the National Scientific and Technical Research Council (CONICET), Buenos Aires, Argentina; ^3^Laboratory of Radioisotopes, School of Pharmacy and Biochemistry, University of Buenos Aires, Buenos Aires, Argentina

**Keywords:** histamine H4 receptor, breast cancer, gastrointestinal cancer, melanoma, lung cancer, bladder cancer, anticancer treatment

## Abstract

Cancer is a leading cause of death in both developed and developing countries. Although advances in cancer research lead to improved anti-neoplastic therapies, they continue to have unfavorable outcomes, including poor response and severe toxicity. Thus, the challenge for the new therapeutic approaches is to increase anti-tumor efficacy by targeting different molecules encompassed in the tumor and its microenvironment, as well as their specific interactions. The histamine H4 receptor (H4R) is the last discovered histamine receptor subtype and it modulates important immune functions in innate and in adaptive immune responses. Several ligands have been developed and some of them are being used in clinical trials for immune disorders with promising results. When searched in The Cancer Genome Atlas (TCGA) database, human H4R gene was found to be expressed in bladder cancer, kidney cancer, breast cancer, gastrointestinal cancers, lung cancer, endometrial cancer, and skin cancer. In the present work, we aimed to briefly summarize current knowledge in H4R’s pharmacology and in the clinical use of H4R ligands before focusing on recent data reporting the expression of H4R and its pathophysiological role in cancer, representing a potential molecular target for cancer therapeutics. H4R gene and protein expression in different types of cancers compared with normal tissue as well as its relationship with patient prognosis in terms of survival will be described.

## Introduction

Cancer is among the leading causes of death in both developed and developing countries. In 2018, 18.1 million new cancer cases and 9.6 million cancer deaths including non-melanoma skin cancer were estimated (Bray et al., 2018). Although advances in cancer research lead to improved anti-neoplastic therapies, they continue to have unfavorable outcomes, including poor response and severe toxicity. Thus, the challenge for the new therapeutic approaches is to increase anti-tumor efficacy by targeting different molecules encompassed in the tumor and its microenvironment, as well as their specific interactions.

Remarkable advances have been made in the understanding of the interactions between immune system and tumor progression, which paralleled with the development of effective immunotherapies, including the immune-checkpoint in a variety of neoplasias (Motz and Coukos, 2013).

Histamine is a biogenic amine with numerous immunomodulatory roles (Gantner et al., 2002; Medina and Rivera, 2010; Stark, 2013), being the major mediator of the acute and chronic inflammatory and the immediate hypersensitivity responses (Jutel et al., 2009; Stark, 2013). Histamine acts through four different receptor subtypes: H1, H2, H3, and H4 receptors (H1R, H2R, H3R, and H4R) (Medina and Rivera, 2010; Stark, 2013). The H4R is the last discovered subtype and is mainly expressed in cells of the immune system, such as mast cells, basophils, eosinophils, monocytes, dendritic cells, T lymphocytes, and Natural Killer (NK) cells (Stark, 2013; Deiteren et al., 2015; Thurmond, 2015). Its functional expression is further described in different types of tumors (Medina and Rivera, 2010; Medina et al., 2013; Cai et al., 2014; Massari et al., 2017, 2019).

IL-2 immunotherapy and histamine dihydrochloride were used in numerous clinical trials for the treatment of solid tumors and hematopoietic cancers with promising outcomes. Histamine preserved the activity of T lymphocytes and NK cells by inhibiting the NADPH oxidase-induced formation of ROS by monocytes/macrophages through the H2R, further supporting the immunomodulatory role of histamine (Hellstrand et al., 2000; Agarwala et al., 2002; Asemissen et al., 2005; Brune et al., 2006; Perz and Ho, 2008; Martner et al., 2010; Berry et al., 2011; Buyse et al., 2011; Yang and Perry, 2011; Rydström et al., 2017; Sander et al., 2017; Kiffin et al., 2018). It is important to highlight that histamine dihydrochloride, used in combination with IL-2, has been approved in Europe for the treatment of adults with acute myeloid leukemia (AML) (Rydström et al., 2017).

Cancer is a complex and highly heterogeneous disease, representing a challenge for the discovery of biomarkers to predict treatment response and/or the prognosis of the pathology, and for the development of targeted treatments. Drug discovery could be improved by sharing genomic data sets, which could lead to the identification of promising novel biomarkers, especially where therapeutic options are currently lacking. Clinical validation of those novel cancer biomarkers and treatment strategies is urgently needed.

Although vast literature can be found describing the use of H1R and H2R ligands in preclinical and even in clinical studies (Massari et al., 2019), less data is available regarding the role of H4R in cancer.

In the present work, we aimed to briefly summarize current knowledge on H4R pharmacology and H4R ligands clinical use before focusing on recent data reporting the expression of H4R and its pathophysiological role in cancer, representing a potential molecular target for cancer therapeutics. Furthermore, when available, the relationship between the H4R expression and patient’s prognosis will be described in different types of cancers.

## H4R Pharmacology and Clinical Studies

**FIGURE 1:**
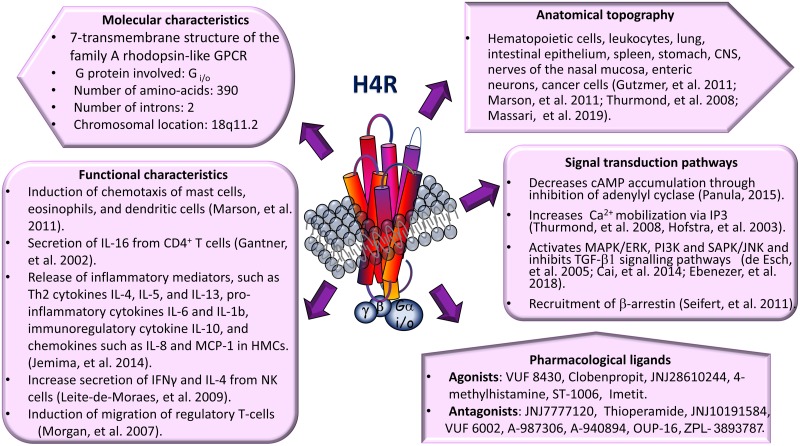
Main molecular and biochemical characteristics of histamine H4 receptor (H4R). GPCR, G protein-coupled receptor; MCP-1, monocyte chemoattractant protein 1; HMCs, human mast cells; IFNγ, interferon-gamma; NK, natural killer; CNS, central nervous system; cAMP, cyclic Adenosine monophosphate; IP3, inositol trisphosphate; TGF-β1, transforming growth factor beta 1.

At the beginning of the millennium, the expression of H4R was described independently by different academic and industry scientific groups. H4R is a Gαi/o coupled receptor, it is predominantly expressed in cells of the immune system and it is particularly involved in immunomodulatory pathways. The main characteristics of H4R are summarized in Figure 1.

Soon after its discovery, the pharmaceutical industry attempted to identify the pharmacological profile of H4R in order to develop selective H4R ligands (agonists or antagonist). The discovery of the first selective and potent H4R antagonist JNJ7777120 by Johnson & Johnson Research and Development (now Janssen Pharmaceuticals, Inc.) was essential for evaluating the role of H4R in pathophysiology, including immune reaction-associated pruritus and inflammation (Thurmond et al., 2017). This compound is the reference tool for studying the functional activity of H4R and it has been employed in several *in vitro* and *in vivo* studies since its development. However, far from being an ideal antagonist, it has drawbacks such as short *in vivo* half-life and toxicity, which prevented further clinical studies. Few clinical data are reported on H4R ligands. Only H4R antagonists are being tested in clinical settings for their potential therapeutic application in immune-related disorders. Recently, the compound JNJ39758979 was developed as a more potent and selective H4R antagonist than JNJ7777120 and it also shows preclinical anti-inflammatory and antipruritic effects (Thurmond et al., 2014, 2017). JNJ39758979 exhibited good preclinical and phase 1 safety in healthy (non-allergic) volunteers and, therefore, progressed to clinical trials in humans (Kollmeier et al., 2014; Thurmond et al., 2014). Although it was effective in reducing pruritus and improving eczema in Japanese adult patients with atopic dermatitis, it showed agranulocytosis, a drug associated, life-threatening side effect, which precluded its clinical use (Thurmond et al., 2014; Murata et al., 2015). Drug-induced agranulocytosis appeared to be an off-target effect, likely intrinsic to the chemical structure of the compound (Thurmond et al., 2014; Murata et al., 2015).

JNJ39758979 was also tested in a phase 2a trial in adults with uncontrolled asthma without reaching the primary efficacy endpoint. However, findings suggest a potential effectiveness in eosinophilic asthma patients. It is important to highlight that no serious adverse events were reported in this study (Kollmeier et al., 2018).

To overcome the side effect associated with agranulocytosis, another H4R antagonist with a different chemical structure was developed under the name of toreforant (JNJ38518168). This compound has been safely administered in clinical studies in patients with rheumatoid arthritis, asthma, and psoriasis (Thurmond et al., 2017). Although toreforant (30 mg per day) failed to improve uncontrolled, eosinophilic asthma (Kollmeier et al., 2018), its efficacy at 30 and 60 mg was greater than placebo in patients with moderate-to-severe psoriasis, but it did not meet the predefined success criterion (Frankel et al., 2018). In addition, in a phase 2 study, toreforant (100 mg per day) reduced rheumatoid arthritis signs and symptoms. However, in phase 2b, no significant improvement was observed (Thurmond et al., 2016).

A recent phase 2a clinical trial was carried out with the selective H4R antagonist ZPL-3893787, administered orally in patients with moderate to severe atopic dermatitis. This compound further reinforced the antipruritic and anti-inflammatory effects of H4R antagonists (Werfel et al., 2018; Schaper-Gerhardt et al., 2019).

As far as we know, until now the clinical use of H4R ligands in other diseases, such as cancer, is missing and warrants further investigations.

## Clinicopathological Characteristics of H4R in Gastrointestinal Cancers

Gastrointestinal cancer comprises the development of a neoplastic disease in the organs that form the digestive system including esophagus, gallbladder, biliary tract, liver, pancreas, stomach, small intestine, bowel (large intestine or colon and rectum), and anus. According to the World Health Organization, colorectal cancer (CRC) and gastric cancer (GC) are the third and the sixth most common cancers. In addition, CRC, GC, and liver cancer are the second, third, and fourth most common causes of cancer death, respectively (Ferlay et al., 2018). Gastrointestinal malignancies varied in etiology, prognosis, clinical course, management strategies, and the mainstay of treatment involved surgery and chemotherapy (Athauda et al., 2019).

We summarize existing preclinical evidences targeting H4R for gastrointestinal cancer treatment in Table 1.

**Table 1:** Preclinical evidence targeting H4R for gastrointestinal cancer treatment.

Cancer type	Experimental model/cells	Outcome	References
Esophageal cancer	Human TE-1, TE-2, TE-3, and TE-13 ESCC cell lines	H4R agonist (4-methylhistamine): ↓ proliferation; ↓ invasion; arrest in G0/1 phase; ↓ TGF-β1 expression in a MAPK- and ACSS2-dependent manner.	He et al., 2018
	TE-2 xenograft in mouse	4-methylhistamine: ↓ xenograft tumor growth *in vivo* and ↑ survival of tumor-bearing animals.	He et al., 2018
Gastric cancer	Human AGS cell line	Histamine and clobenpropit: induce G0/G1 phase cell cycle arrest.	Zhang et al., 2012
Colon cancer	Human HT29, Caco-2, and HT116 CRC cell lines	H4R antagonist (JNJ7777120): ↓ proliferation; ↓ COX-2 and VEGF expression.	Cianchi et al., 2005
	Inflammation-associated CRC in mice	H3R antagonist and H4R agonist (clobenpropit): ↓ CRC carcinogenesis.	Tanaka et al., 2016
	Colo-320, Mock-Lovo, and H4R-Lovo CRC cells	H4R activation: ↑ expression of p21^Cip1^ and p27^Kip1^ proteins, arrest in G0/1 phase through cAMP/PKA-dependent signaling.	Fang et al., 2011
Pancreatic cancer	Human Panc-1 cell line	Clobenpropit: ↓ proliferation.	Cricco et al., 2004
	Human Panc-1, MiaPaCa-2 and AsPC-1 cell lines	Clobenpropit: ↓ proliferation.	Paik et al., 2014
	Panc-1 xenograft mouse	Clobenpropit and gemcitabine: ↓ tumor growth.	Paik et al., 2014
Liver cancer	Human CHOL cell lines	Clobenpropit: ↓ proliferation by Ca^2+^-dependent pathway and altered EMT and invasion.	Meng et al., 2011
		Overexpression of H4R: ↓ proliferation.	
	Mz-ChA-1 xenograft mouse	Clobenpropit: ↓ tumor growth.	Meng et al., 2011


**ESCC, esophageal squamous cell carcinoma; CHOL, cholangiocarcinoma; ACSS2, acetyl-coenzyme A synthetase 2; CRC, colorectal cancer; EMT, epithelial-mesenchymal transition*.*

Cancer of the esophagus, including adenocarcinoma and esophageal squamous cell carcinoma (ESCC) subtypes, is the seventh most frequent cancer worldwide and the sixth most common cause of cancer death with 572,034 new diagnoses and 508,585 deaths in 2018 (Bray et al., 2018; Walker and Underwood, 2018). Both histological subtypes exhibit a poor prognosis with an overall 5-year survival smaller than 20%, especially in patients with advanced tumor stages (Bray et al., 2018; Xie and Lagergren, 2018). Therefore, as for other types of cancer, prevention and early detection are crucial in reducing cancer burden. Surgical resection and highly morbid chemotherapy and radiotherapy are the mainstay treatments for esophageal cancer, which are still associated with recurrence (Kato and Nakajima, 2013; Chen et al., 2019). For this reason, further research is necessary to develop more effective therapeutics.

Several differentially expressed genes in tumor samples compared to non-tumorigenic tissue could be identified using data of public profiling arrays. The Cancer Genome Atlas (TCGA) is a publicly available database that shows the most important genomic changes in tumor tissue and matched normal tissues from more than 11,000 patients of 33 types of cancers. Therefore, it notably contributes to accelerate the understanding of the molecular basis of cancer (Chandrashekar et al., 2017). Transcriptomic data available in TCGA project were analyzed using web tools, including UALCAN, cBioPortal, and Firebrowse portals. UALCAN and Firebrowse obtain RNA-seq data from primary solid tumors and normal tissue from TCGA. Using these data, we investigated differences in the H4R gene expression levels and searched potential correlations of H4R gene expression and overall survival (OS).

**FIGURE 2:**
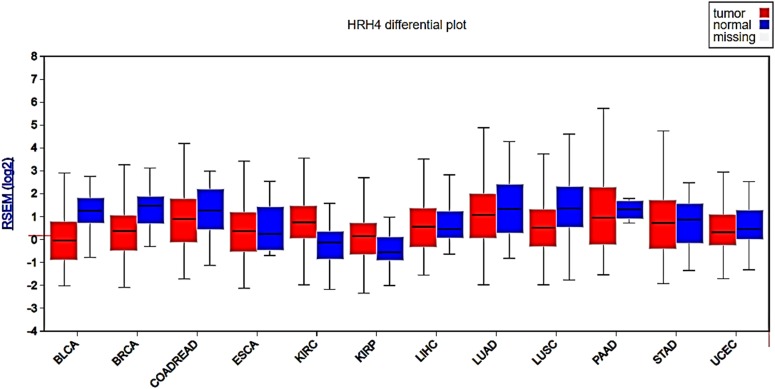
Analysis of H4R gene expression in different types of human cancers. Box plots show H4R expression levels measured as log2 RSEM (RNA-Seq by Expectation Maximization) from tumor (red box) and normal tissue (blue box). RNA-seq data was obtained from TCGA samples and analyzed using Firebrowse web resource (http://firebrowse.org, Deng et al., 2017). BLCA, bladder urothelial carcinoma; BRCA, breast invasive carcinoma; COADREAD, colorectal adenocarcinoma; ESCA, esophageal carcinoma; KIRC, kidney renal clear cell carcinoma; KIRP, kidney renal papillary cell carcinoma; LIHC, liver hepatocellular carcinoma; LUAD, lung adenocarcinoma; LUSC, lung squamous cell carcinoma; PAAD, pancreatic adenocarcinoma; UCEC, uterine corpus endometrial carcinoma. Additional statistical parameters are displayed in Supplementary Figure S2.

According to the TCGA information, H4R gene expression is significantly higher in primary tumors when compared with its expression in normal tissue (Figure 2 and Table 2). In agreement with these observations, it was recently reported the expression of H4R not only at the mRNA but also at the protein level in ESCC. Patients with high protein expression of H4R in tumor cells exhibited a larger primary tumor size and a greater number of lymph node metastases than those with lower expression. The stage of a cancer (I–IV), based on the AJCC (American Joint Committee on Cancer) pathologic tumor stage information, describes the size of a tumor and whether it has spread into nearby lymph nodes or to other organs (Amin et al., 2017). Kaplan–Meier curves for patients with AJCC stage III cancer (57 patients) were further stratified by H4R expression. The median survival rate was higher for patients with reduced expression of H4R (He et al., 2018). In contrast, no difference in overall survival (OS) was observed in TCGA probably because Kaplan–Meier curves include all stages and both histological esophageal cancer types (Table 2).

**Table 2:** Differential expression of H4R in cancer.

	Cancer type	*H4R* expression in tumor vs. normal tissue^∗^	Overall survival with high *H4R* expression^∗^	Fold change of *H4R* expression in tumor vs. normal tissue^∗∗^	Alteration frequency of *H4R* gene^#^
Gastrointestinal cancer	Colon cancer	**↓** (*P* < 0.05)	NS	0.772	0.78%
	Pancreatic cancer	NS	**↑** (*P* = 0.05)	ND	9.68%
	Esophagus cancer	**↑** (*P* < 0.05)	NS	1.090	8.06%
	Stomach cancer	**↓**st2, **↑** st3 (*P* < 0.01)	NS	0.950	5.44%
	Liver cancer	**↑** (*P* < 0.05)	NS	1.080	0.45%
Urogenital cancer	Bladder urothelial carcinoma	**↓** (*P* < 0.05)	NS	0.406	2.91%
	Kidney renal clear cell carcinoma	**↑** (*P* < 0.001)	**↓** (*P* = 0.034)	1.840	0.74%
	Kidney renal papillary cell carcinoma	NS	NS	1.630	0.34%
	Uterine corpus endometrial carcinoma	↓ (*P* < 0.01)	**↓** (*P* = 0.0037)	0.909	2.19%
Lung cancer	Lung adenocarcinoma	NS	NS	0.836	3.24%
	Lung squamous cell carcinoma	↓ (*P* < 0.01)	NS	0.577	4.31%
Breast cancer	Breast invasive carcinoma	↓ (*P* < 0.001)	NS	0.465	1.27%


**^∗^http://ualcan.path.uab.edu Additional information is displayed in Supplementary Figure S1. ^∗∗^http://firebrowse.org Additional information is displayed in Supplementary Figure S2. ^#^http://www.cbioportal.org Additional information is displayed in Supplementary Figure S3. NS, not significant; ND, not determined*.*

Preclinical data show that H4R activation using the H4R agonist 4-methylhistamine not only blocked cell cycle, proliferation, and invasion of ESCC cells, but also reduced TE-2 tumor xenografts and increased the survival of tumor-bearing mice (Table 1), which suggests that H4R could be a target for ESCC treatment (He et al., 2018).

At early stages gastric cancer is almost a curable malignancy; however, most of the patients are diagnosed at advanced stages, which shows a poor 5-year survival rate (Dicken et al., 2005; Bray et al., 2018). While treatment modalities include surgery and chemotherapy (Song et al., 2017), little is known about the participation of H4R in gastric cancer.

According to TCGA data, H4R expression level is reduced in primary tumor samples compared with normal tissue (Figure 2 and Table 2). Previous data showed, in gastric cancer samples obtained from 131 Chinese surgical patients, a down-regulation of *H4R* in gastric carcinomas especially for cancer stage 3 and 4 compared to the adjacent normal tissue, which suggest that H4R plays a role in histamine-mediated growth control of gastric cancer cells. Furthermore, authors demonstrated that deletion and downregulation of *H4R* gene take place in the progression but not the initiation of stomach cancer (Zhang et al., 2012).

As it was observed in ESCC, H4R agonists (histamine and clobenpropit) reduced growth and induced G0/G1 cell cycle arrest in AGS cell line (Zhang et al., 2012), (Table 1).

For both sexes combined CRC is the third most commonly diagnosed and the second most common cause of death by cancer worldwide, with extensive geographical variation in incidence and mortality (Bray et al., 2018; Pilleron et al., 2019). Although frequently combined with colon cancer (as CRC), rectal cancer accounts for one-third of CRCs and is particularly challenging with regard to treatment strategies and outcomes (Tamas et al., 2015). Treatment for CRC is based largely on the cancer stage (extent) and the implementation of novel treatment modalities have significantly improved the management of CRC, with surgery remaining as the main pillar. Radiation and chemotherapies may also be used before or after surgery (Banerjee et al., 2017; Ratnapradipa et al., 2017; São Julião et al., 2017), while immunotherapy arises as a novel promising therapeutic approach for this pathology (Gutting et al., 2018). The role of histamine and histamine receptors in this cancer subtype has been more extensively investigated than in gastric, esophageal or liver cancer (reviewed in Medina and Rivera, 2010; Massari et al., 2019).

By means of TCGA genomic data, we demonstrated that *H4R* gene expression is significantly reduced in primary colon tumors compared with normal colon tissue (Figure 2 and Table 2, Massari et al., 2019). Rectal cancer samples followed the same expression pattern. Accordingly, it was previously demonstrated the protein expression of H4R in human CRC cell lines and tissue that further confirmed the down-regulation of H4R expression observed in tumor samples compared to normal colonic tissue (Boer et al., 2008). In line with these results, Fang et al. (2011), showed reduced expression of H4R in advanced CRC compared with those in an initiating stage. These authors also showed that H4R activation using agonists in CRC cell line overexpressing H4R resulted in reduced growth and progression (Fang et al., 2011) (Table 1). On the other hand, a previous study showed that JNJ7777120 inhibited histamine-induced cell growth of human CRC cell lines (Cianchi et al., 2005). However, a newer study showed again that H4R activation with the H3R antagonist/H4R agonist clobenpropit reduced CRC inflammation-associated carcinogenesis (Tanaka et al., 2016). Further *in vitro* and *in vivo* studies with specific H4R compounds should be designed to confirm the anti-tumoral effects of H4R agonists.

In addition, it was reported that histidine decarboxylase (HDC) deficiency promoted inflammation-associated CRC. Exogenous histamine administration induced differentiation of CD11b+Gr-1+ immature myeloid cells and suppressed their ability to support the tumor growth (Yang et al., 2011). Furthermore, more recently, Gao et al. (2017), showed that histamine produced by the gut microbe hdc+ *Lactobacillus reuteri*, histamine-producing probiotic, reduced chronic intestinal inflammation and colorectal tumorigenesis in Hdc-/- mice. Administration of *L. reuteri* and not of an isogenic HDC-deficient *L. reuteri* mutant that was unable to generate histamine, suppressed carcinogenesis, cancer-associated cytokines, and decreased the relative number of splenic CD11b+Gr-1+ immature myeloid cells, which confirmed the potential antitumorigenic effect of histamine (Gao et al., 2017).

Liver cancer is the sixth most commonly diagnosed cancer and the fourth leading cause of cancer death worldwide in 2018, with about 841,000 new cases and 782,000 deaths annually (Bray et al., 2018). Primary liver cancer includes hepatocellular carcinoma (HCC) (comprising 75–85% of the cases) and intrahepatic cholangiocarcinoma (CHOL, comprising 10–15% of the cases) as well as other rare types. Chronic infection with hepatitis B or C virus, alcohol abuse, liver cirrhosis, aflatoxin-contaminated foodstuffs are main risk factors for HCC (Thun et al., 2010; Bray et al., 2018). At diagnosis, almost 70% of the patients have only access to a palliative treatment (Llovet et al., 2016). Treatments include surgery, sorafenib as first-line therapy (Llovet et al., 2008) and regorafenib as second-line treatment, both drugs with modest OS benefit (Bruix et al., 2017; Roth and Decaens, 2017). Prognosis of CHOL is also considered dismal and the majority of patients are diagnosed at late stages of the disease. Surgical treatment, whenever possible, is the only potentially curative therapeutic option for CHOL (Blechacz, 2017).

Few studies show the participation of histamine in biological processes associated to liver carcinogenesis or demonstrated the expression of the four histamine receptor subtypes. Histamine seemed to produce a dual effect on proliferation in both HCC and CHOL depending on the receptor involved (for review Massari et al., 2019). Analyses of TCGA cancer genomics data show a modest but significant increase in the expression of H4R levels in HCC primary tumors compared with normal tissue (Figure 2 and Table 2). Immunohistochemical studies in patient samples are needed to confirm these differences at the H4R protein level. Similarly, H4R gene expression in CHOL primary tumors (*n* = 36) is slightly increased compared to normal tissue (*n* = 9) (Massari et al., 2019). In line with these observations, it was shown that H4R immunoreactivity in human CHOL specimens is increased compared with non-malignant tissues (Meng et al., 2011).

Consistent with the results in CRC and pancreatic cancer models, the H3R antagonist/H4R agonist clobenpropit inhibited human CHOL proliferation, decreasing tumor invasion and growth through disruption of the epithelial-mesenchymal transition (EMT) using *in vitro* and *in vivo* model systems. To confirm the exclusive participation of the H4R in these processes, studies were performed in cells with genetic knockdown of the H3R and overexpression of H4R (Meng et al., 2011). Further studies are necessary to fully understand the role of histamine and H4R in liver cancer progression.

Pancreatic cancer remains the seventh leading cause of cancer-related mortality in both males and females (Bray et al., 2018). Although surgery remains the only curative treatment for pancreatic cancer, most of the patients have unresectable disease and approximately 80% patients who undergo curative intent surgery ultimately relapse. The standard of care is chemotherapy; however, it constitutes a relatively chemotherapy-resistant cancer. All of these characteristics, together with the propensity to metastasize early in the course of disease, predict its poor prognosis (Diab et al., 2018; Young et al., 2018).

As in liver cancer, histamine seems to produce a dual effect on proliferation depending on the histamine receptor subtype that is activated. The presence of H4R was reported in three human pancreatic cell lines (Panc-1, MiaPaCa-2, and AsPC-1) (Cricco et al., 2008; Paik et al., 2014). In these cell lines, clobenpropit inhibited cell proliferation and cell migration, disrupting ETM. Combination therapy of clobenpropit and gemcitabine reduced tumor growth and enhanced apoptosis in Panc-1 xenograft induced in mice (Paik et al., 2014).

Although not significant, probably due to the reduced number of samples, *H4R* gene expression in pancreatic primary tumors (*n* = 178) seems to be decreased compared to normal tissue (*n* = 4) by means of TCGA database and UALCAN web portal (Table 2 and Supplementary Figure S1). Immunohistochemical studies in a large number of patient specimens are still missing and should be investigated. It is important to highlight that high expression of H4R in tumor specimens was associated with increased OS in pancreatic cancer (Table 2). In contrast, no significant difference in OS between low/medium H4R expression level and high H4R expression level tumor samples was observed using TCGA data in other gastrointestinal cancers (Table 2).

Described evidence indicate that H4R expression is modulated in different gastrointestinal cancers. The expression of the H4R seems to be reduced in human CRC and GC and increased in ESCC, CHOL, and liver cancer compared to normal tissue. Also, it could vary between early and advanced stages, which suggests the participation of H4R in the carcinogenesis process. This deserves further investigation in order to deeply understand the role of H4R in cancer development and progression in view of identifying a novel therapeutic target or even a useful prognostic biomarker.

## H4R Expression in Urinary Tract Cancers

The predominant urinary tract malignancies are bladder and kidney cancer (Yaxley, 2016). Despite advances in prevention, detection, and therapeutics, the incidence of age-related cancers of the urinary system is probably increasing as a result of population aging (Golabek et al., 2015; Bray et al., 2018).

Bladder cancer is the most common malignancy in the urinary system and is the 10th most common form of cancer worldwide. Interestingly, bladder cancer is more frequent in men than in women, with respect to incidence and mortality rates (Bray et al., 2018). Most cases are bladder urothelial carcinomas (BLCA), which often appear as superficial papillary tumors with good prognosis and surgery as a primary treatment. However, muscle-invasive carcinomas are presented in about thirty percent of cases, having a higher risk of metastasis development and may need cystectomy and adjuvant chemotherapy (Jimenez-Guerrero et al., 2018; Li et al., 2018).

The most common type of kidney cancer is called renal cell carcinoma. An estimated 65,340 cases were expected to have been diagnosed in 2018 and an estimated 14,970 to have died of this cancer (Bray et al., 2018). TCGA analysis shows two subtypes called kidney clear cell carcinoma (KIRC), which represents approximately 92 percent of such cases, and papillary carcinoma (KIRP). KIRC is associated with resistance to radio and chemotherapy, and with poor OS, particularly for patients with late-stage disease. As in other types of cancers, early detection of kidney cancer favors an effective treatment. Standard surgical resection is used for localized tumors. Patients with metastatic cancer benefit from chemotherapy based on the tyrosine kinase inhibitor (e.g., cabozantinib, sunitinib) (XIao et al., 2018; Yu et al., 2018).

As previously indicated, numerous studies show that histamine and H4R are involved in cell proliferation, a key event in tumor development and progression, in different types of tumors. However, to the best of our knowledge, a comprehensive investigation of whether H4R is involved in the carcinogenesis of the urinary system has not yet been performed.

Similar to what was previously demonstrated in CRC, TCGA data showed attenuated *H4R* gene expression in human BLCA specimens compared to normal tissue (Figure 2, Massari et al., 2019). No significant differences were observed between low/medium or high expression at least in OS (Table 2). On the other hand, as it was observed in ESCC and liver cancer, KIRC and KIRP exhibited increased *H4R* gene expression compared to normal tissue (Figure 2 and Table 2). KIRC patients with high expression levels of H4R show a significant reduced OS compared with the ones with low/medium expression (Table 2). Future research should study the potential association of H4R expression and clinicopathological characteristics in human specimens of tumors of the urinary tract, of different stages and histological types, to confirm the potential role of H4R as a prognostic biomarker of these malignancies.

In addition, studies in experimental tumor models are necessary to determine the potential therapeutic value of H4R in bladder and/or kidney cancers.

## H4R Expression in Gynecological and Breast Cancer

Breast cancer is the most frequently diagnosed neoplasia and the leading cause of cancer death in women worldwide. Incidence rates of breast cancer have been continuously rising in most countries (Bray et al., 2018). The cancer of the breast is a heterogeneous disease, showing different histological types, molecular profile and clinical response to therapy. Depending on the presentation and the molecular and histological characteristics, treatments include surgery, radiation therapy, chemotherapy, and targeted molecular treatments (Nagini, 2017).

Histamine plays a critical role in physiological conditions of the mammary gland as well as in breast cancer. Numerous studies reported the expression of the four histamine receptors and show that all of them are implicated in biological processes associated to breast cancer (Martinel Lamas et al., 2015c; Massari et al., 2019). Since the first description of the H4R, new functions for this receptor are continuously discovered.

The first description of H4R in cancer was reported in breast tumors. H4R is expressed at the protein and mRNA level in malignant lesions of the human mammary gland and in MDA-MB-231 and MCF-7 breast cancer cell lines (Medina et al., 2006, 2008). These results are consistent with findings from TCGA data in a large number of human samples. *H4R* gene is expressed in primary tumors and its expression is significantly reduced compared with normal tissue (Massari et al., 2019, Figure 2). However, no significant changes were observed between low/medium or high expression at least in OS (Table 2).

Further supporting the critical role of H4R in breast cancer development and progression, He and coworkers demonstrated the presence of polymorphisms of the *H4R* gene (rs623590, rs11662595, and rs1421125 genotypes of *H4R* gene) in Chinese Han population, which were associated with the risk of developing breast cancer and the malignant degree of the tumor (He et al., 2013).

A summary of preclinical evidences targeting H4R in breast cancer is depicted in Table 3. All the studies, using different H4R ligands and also genetic down-regulation of H4R, demonstrated that the principal receptor subtype involved in the histamine-induced reduction of proliferation was the H4R (Medina et al., 2008; Martinel Lamas et al., 2013). The *in vivo* administration of histamine or H4R agonists (e.g., JNJ28610244) diminished the tumor growth of human triple negative breast cancer (TNBC) developed in immune-deficient nude mice with MDA-MB-231 cells (Martinel Lamas et al., 2013, 2015b). On the other hand, tumor doubling time was not significantly modified while mean survival was reduced after the treatment with the H4R antagonist JNJ10191584 (Martinel Lamas et al., 2013).

**Table 3:** Preclinical evidence targeting H4R for the treatment of melanoma, breast, lung, and testicular cancers.

Cancer type	Experimental model/cells	Outcome	References
Lung Cancer	Human NSCLC cell lines and xenograft NSCLC tumors	H4R agonist (4-methylhistamine): ↓tumor volume, ↑ survival, ↑ E-cadherin and ↓ vimentin (↓ EMT progress).	Cai et al., 2014
Breast cancer	Human MDA-MB-231 and MCF-7 cell lines	H4R agonists (histamine, clobenpropit, VUF8430): ↓ proliferation; ↑ apoptosis; ↑ senescence; cell cycle arrest.	Martinel Lamas et al., 2013; Medina et al., 2008, 2011
	MDA-MB-231 xenograft in mouse	H4R agonists (histamine, JNJ28610244): ↓ tumor volume; ↓ angiogenesis. Histamine: ↑ survival.	Martinel Lamas et al., 2013, 2015a
Melanoma	Human WM35 and M1/15 cell lines	H4R agonists (Clozapine, VUF8430): ↓ proliferation, ↑ senescence, inhibited forskolin-induced cAMP levels in M1/15, increased phosphorylation levels of ERK1/2 in both cell types.	Massari et al., 2013
	Human 1205Lu cell line	H4R agonists (histamine, Clozapine, JNJ28610244): ↓ proliferation, ↑ senescence, ↑ melanogenesis.	Massari et al., 2017
	M1/15 xenograft in mouse	Histamine and clozapine: ↓ tumor volume, ↑ survival.	Massari et al., 2013
	1205Lu xenograft in mouse	H4R agonists (histamine, Clozapine, JNJ28610244): ↓ tumor growth, ↓ mitotic index, and PCNA in tumor, ↓ metastatic spread, and angiogenesis.	Massari et al., 2017
Testicular cancer	Mouse MA-10 cell line	H4R agonists (JNJ28610244 and VUF8430): ↓ proliferation, VUF8430: ↓ LH/hCG-stimulated cAMP production and ↓progesterone production.	Abiuso et al., 2014
	Rat R2C cell line	H4R agonist (VUF8430): ↓ proliferation, ↓ steroidogenesis, ↓ pro-angiogenic capacity.	Abiuso et al., 2018


**EMT, epithelial to mesenchymal transition; LH/hCG, luteinizing hormone/choriogonadotropin receptor; NK, natural killer; NSCLC, non-small cell lung cancer; OTSCC, oral tongue squamous cell carcinoma; PCNA, proliferating cell nuclear antigen*.*

In the same model, histamine administration was demonstrated to potentiate ionizing radiation and doxorubicin therapies (Martinel Lamas et al., 2015a,b,d).

Considering the pleiotropic nature of histamine actions and its numerous immunomodulatory roles, recently it was demonstrated a novel role of H4R in the antitumor immunity of breast cancer (Sterle et al., 2019). The study was performed in a model of TNBC developed orthotopically with 4T1 cells in H4R KO compared with wild type mice and no treatments were used. Mice lacking H4R show reduced tumor growth and percentage of CD4+ tumor-infiltrating T cells together with increased infiltration of natural killer (NK) cells (Sterle et al., 2019). These findings highlight the critical interplay between tumor cells and host immune cells, which could determine the clinical therapeutic outcomes of a pharmacological compound. Therefore, it is important to validate the potential use of H4R ligands in immunocompetent hosts.

Gynecologic cancer is the fourth most common cancer in women and 1 in every 20 women is estimated to develop gynecologic cancer in their lifetime. The main types are ovarian, cervical, uterine, vaginal, and vulvar. Each of these cancers have different disease evolution, prevention strategies, and therapies.

Cancer of the corpus uteri is usually indicated as endometrial cancer, which is a common gynecological malignancy. Although the overall prognosis is relatively good, high-grade tumors tend to recur. Standard treatment consists of hysterectomy and bilateral salpingo-oophorectomy while combination of surgery and chemotherapy is employed in advanced disease (Bartosch et al., 2011; Amant et al., 2018).

It was previously reported that histamine and polyamines are increased in human ovarian, cervical and endometrial cancer in comparison with normal tissues (Chanda and Ganguly, 1995). However, little is known about the expression of histamine receptors and their role in tumorigenesis.

Although mRNA expression of H1R and H2R was shown, the expression of H3R and H4R was hardly detected in HEC-1 endometrioid adenocarcinoma cells. Histamine through the H1R increased aldehyde dehydrogenase 1 high population, a marker of cancer-initiating cells, which was associated to histamine-induced invasiveness and drug resistance (Wang et al., 2014).

UALCAN and Firebrowse analyses of TCGA data indicated that *H4R* expression levels are reduced in samples of uterine corpus endometrial carcinoma (UCEC) compared to non-malignant tissue, in particular in endometrioid cancer, the most common type of endometrial cancer (Figure 2 and Table 2). Furthermore, patients with tumors showing low/medium *H4R* expression presented a higher OS compared to those with high expression levels (Table 2).

Further studies are needed to validate the potential of H4R targeted therapy in the treatment of breast and gynecological cancers.

## H4R Expression and Lung Cancer

Worldwide, lung cancer is the most commonly diagnosed cancer and the leading cause of cancer death in both sexes combined (Bray et al., 2018). Non-small cell lung cancer (NSCLC) is the most common form of lung cancer and comprises adenocarcinoma (LUAD), squamous cell carcinoma (LUSC), and large cell carcinoma (Stoyanov et al., 2012). Therapeutic approaches strongly depend on cancer stage. Surgery is the recommended treatment for patients with stages I–II NSCLC (Vansteenkiste et al., 2014). For patients with clinical stage I NSCLC with unresectable lesions, high-dose stereotactic body radiation therapy is recommended. The standard of care for patients with locally advanced NSCLC is thoracic radiotherapy with the concurrent chemotherapy and for patients with advanced NSCLC who do not fit an approved molecular targeted therapy, the standard first-line treatment remains to be chemotherapy. In addition, early clinical trials with immunotherapy with monoclonal antibodies directed to the PD-1 receptor or its ligand PD-L1 have shown durable responses in about 14–20% of patients with advanced NSCLC (Hirsch et al., 2017). In spite of the advances in novel therapeutics, lung cancer continues leading the rank of cancer death worldwide. Therefore, the development of novel safe and effective molecular targeted therapy is a critical unmet need.

Previous reports suggested the involvement of histamine metabolism and mast cells in the pathogenesis of lung cancer (Stoyanov et al., 2012; Medina et al., 2013; Massari et al., 2019). Histamine plasma levels are decreased in cancer patients (Della Rovere et al., 2006) and the H4R seems to play a critical role in NSCLC progression through preventing EMT progress (Cai et al., 2014).

According to genomic data from TCGA, *H4R* gene expression is significantly reduced in both LUAD and LUSC compared to normal tissue samples (Figure 2 and Table 2), although no significant differences were observed in OS rates in none of the cancer types (Table 2). Likewise, results described in breast cancer, genetic variations of *H4R* gene were found in a large number of Chinese NSCLC patients. In particular, the loss-of-function polymorphism rs11662595, associated to a higher invasive behavior, was linked to the prognosis, the degree of malignancy, and the metastatic potential of NSCLC (Cai et al., 2017).

In this sense, treatment with 4-methylhistamine H4R agonist reduced aggressive potential in both NSCLC cell lines and xenograft NSCLC tumors (Cai et al., 2014), (Table 3).

## H4R Expression in Melanoma and Other Cancers

It is well known that histamine regulates physiological and pathological conditions not only of the skin (Gutzmer et al., 2011; De Benedetto et al., 2015; Lin et al., 2015) but also of numerous organs (Massari et al., 2019). Apart from the cancer types described above, histamine is a crucial modulator of proliferation in other types of tumors, including melanoma and head and neck cancers (Massari et al., 2019).

The incidence and mortality rates of cutaneous melanoma, the deadliest and more aggressive type of skin malignancy, are continuously increasing worldwide (Ingraffea et al., 2012; Bray et al., 2018). Although novel oncogene-targeted therapy and immune checkpoint blockade have shown some efficacy, the clinical benefit in terms of disease-free interval is of only a few months. Therefore, the study of new therapeutic targets is imperative (Block et al., 2015; Marzuka et al., 2015; Bernatchez et al., 2016; Prieto et al., 2016).

Previous findings demonstrated the functional expression of H4R in human melanoma cells and also the presence of H4R in melanoma specimens. H4R expression level in benign lesions of the skin was significantly higher than in malignant tissues. In melanoma samples, H4R immune-expression was inversely correlated with PCNA expression and mitotic index, both proliferation and prognostic markers (Massari et al., 2017).

In agreement with these results, administration of histamine and H4R agonists led to a significant reduction of proliferation and metastatic spread *in vitro* in different human tumor cell lines and *in vivo* in human 1205Lu tumor developed in nude mice (Massari et al., 2011, 2013, 2017), (Table 3).

Last but not least, a recent work in Leydig-cell tumors (LCT), which are rare testicular endocrine tumors shows that H4R agonist VUF8430 treatment inhibited steroidogenesis and proliferation in R2C cells and also reduced pro-angiogenic capacity both *in vitro* and *in vivo*. Furthermore, H4R immunostaining was weak in LCT but moderate/strong in normal prepubertal testes (Abiuso et al., 2014, 2018).

We were not able to compare the *H4R* expression levels between normal and tumor tissue, for melanoma or testicular cancer, due to the absence of non-malignant tissue expression levels using TCGA data.

Further studies are needed to corroborate the potential of H4R as a target for the treatment and also to determine whether the evaluation of H4R expression could be useful as a prognostic biomarker of these malignancies.

A summary of preclinical evidences targeting H4R for the treatment of melanoma and testicular cancer is shown in Table 3.

## Limitations of H4R Translational Research

Numerous preclinical data derived from *in vitro* and *in vivo* experimental models of cancer together with studies of human normal and tumorigenic tissues demonstrated undoubtedly the participation of H4R in cancer progression and suggest the use of H4R ligands in the molecular targeted therapy of cancer. Improvement and validation of H4R targeting compounds should be considered before moving to clinical trials.

One issue is the lack of selectivity of some H4R ligands based, at least in part, in the high structural homology between H3R and H4R. Therefore, many imidazole-containing H3R ligands, have significant affinity for the H4R, including (R)-α-methylhistamine, imetit, and clobenpropit, which all act as agonists at the H4R. The antipsychotic drug clozapine has affinity for other GPCRs but also exhibits H4R agonist activity.

Nonetheless, changing the structure of some compounds increased specificity. This is the case of VUF8430, a full agonist of the H4R with around 30-fold selectivity over the H3R. One of the most selective H4R agonist currently reported is the compound 4-methylhistamine, with more than 100-fold selectivity over the other 3 histamine receptor subtypes (Leurs et al., 2009; Lim et al., 2009; Panula et al., 2015).

As in the case for agonists, the first antagonists for H4R were mostly H3R antagonists. With time, though, more selective inhibitors were developed. Antagonist JNJ7777120, as mentioned before, shows high selectivity for the human, mouse and rat H4R. Also, it has been used extensively as the reference compound to determine the role of H4R antagonism in a variety of experimental animal models of disease (Stark, 2013; Panula et al., 2015). Based on JNJ7777120 structure, diverse compounds have been synthesized such as VUF6002, also known as JNJ10191584. Most of these agonistic and antagonistic compounds were employed to assess the pathophysiological role of H4R in different types of cancer.

Another issue is the fact that different H4R species orthologs could exhibit different pharmacological properties and some H4R ligands exhibited biased signaling for both the G protein and β-arrestin pathways, adding complexity to the H4R pharmacology (Seifert et al., 2011; Nijmeijer et al., 2012; Stark, 2013; Panula et al., 2015).

The evidence mentioned above could explain some discrepancies observed between *in vivo* and *in vitro* findings using H4R compounds. Therefore, the use of a combination of pharmacological tools including agonist and antagonist, genetic modulation of H4R expression and the use of H4R KO mice when possible, could help to better interpret results.

In addition, the contribution of genetic and post-transcriptional variations should be considered in the experimental outcomes with H4R ligands. Human H4R isoforms are the result of alternative splicing. Shorter isoforms do not bind histamine or inverse agonists and at least in heterologous expression systems, they could produce dominant negative effects on the full-length isoform (van Rijn et al., 2008; Panula et al., 2015), suggesting that they may elicit modulatory effects on H4R signaling. The expression of different isoforms in cancer cells and their pathophysiological relevance are still unknown and should be evaluated in future studies.

**FIGURE 3:**
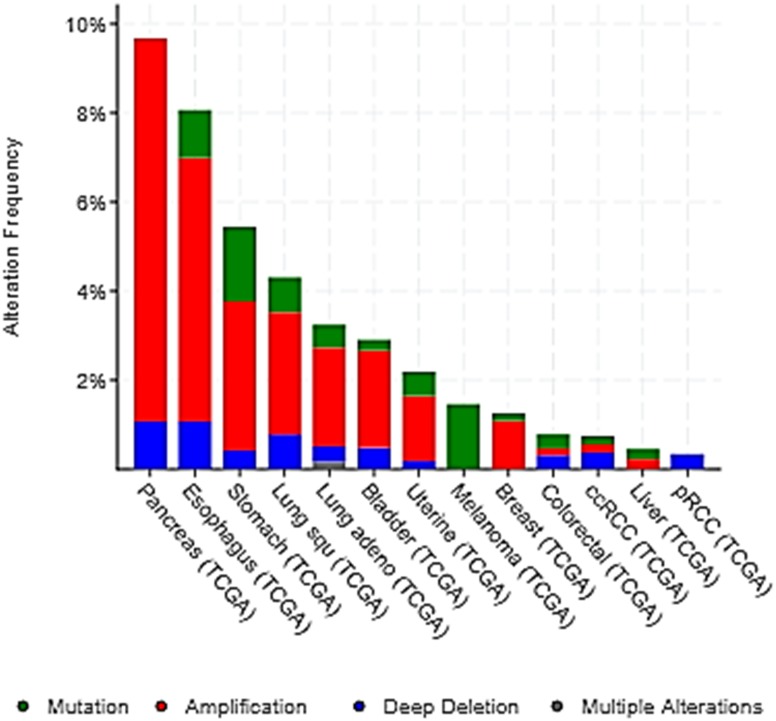
Alteration frequency of H4R gene in the main cancer types. The alterations include deletions (blue), amplification (red), multiple alteration (gray), and mutation (green). Data was obtained from cBIOPortal web resource (http://www.cbioportal.org/, Cerami et al., 2012; Gao et al., 2013). Lung squ, lung squamous cell carcinoma; Lung adeno, lung adenocarcinoma; ccRCC, kidney renal clear cell carcinoma; pRCC, kidney renal papillary cell carcinoma. Additional statistical parameters are displayed in Supplementary Figure S3.

Some polymorphisms of H4R have been reported in cancer, which were associated to malignancy of the disease in a Chinese Han population. Further studies are needed to corroborate these in other populations in order to evidence whether they are ethnicity-based genetic variations that could influence H4R ligands’ effects (He et al., 2013; Micallef et al., 2013; Cai et al., 2017). In addition, the study of the alteration frequency of H4R gene in the main cancer types, with available data from cBioPortal, indicated that H4R gene alterations occurred in different percentage depending on cancer type. Amplifications and mutations comprised the major types of H4R gene alterations (Figure 3). The significance of the genomics alterations of H4R in cancer tissue is still unknown and deserves further investigation.

Last but not least, uncertainties around the selectivity of antibodies used to detect H4R have been reported. Suppliers should improve specificity validation of commercially available antibodies prior to their release to the market. It was recently reported four criteria of which at least one has to be fulfilled to provide evidence for the specificity. They include the use of cells from receptor KO animals, receptor-selective siRNA treated cells, cells transfected with the targeted and non-targeted related receptors or use of various antibodies directed against different epitopes of the receptor (Miyauchi et al., 2009; Beermann et al., 2012; Gutzmer et al., 2012; Seifert et al., 2013).

## Conclusion

According to estimates from the World Health Organization, cancer incidence and mortality are rapidly increasing worldwide. Cancer is expected to rank as the leading cause of death in the 21st century (Bray et al., 2018). Despite the advances in cancer clinical research, including novel molecular targeted therapies and immunotherapy, and the aggressiveness or even multiple therapeutics employed to treat cancer, a high percentage of oncological patients will suffer from overwhelming morbi-mortality, indicating a clear unmet therapeutic need to improve patient survival and quality of life.

A precise and accurate translational cancer research is essential for the development of novel therapeutic agents. In this sense, the use of public genomic data has enormously contributed to expanding our understanding of cancer biology especially in terms of evidencing differences between normal and malignant samples. This could help to develop more specific and less toxic molecular targeted therapies for cancer treatment. In this context, the publicly available TCGA dataset is extremely useful.

The immunohistochemical studies together with the H4R gene expression data from TCGA database and UALCAN and Firebrowse web portals provided consistent evidence showing that H4R is differentially expressed in primary tumors compared with normal tissue, suggesting a crucial role of this histamine receptor subtype in carcinogenesis. The expression of H4R is associated with some clinicopathological features, including cancer stage and survival depending on the cancer type. *H4R* gene expression is reduced in CRC, GC, breast cancer, LSCC, and also bladder urothelial carcinoma, and uterine corpus endometrial carcinoma, while is increased in liver, esophageal and kidney cancers compared to normal tissue. Presented data supported previous findings of H4R expression impairment in cancer, which could influence the histamine-induced growth modulation in cancer cells, suggesting a potential role of abnormal H4R expression in cancer progression. The mechanisms responsible for the differential up or down-regulation of H4R in different cancers are unknown and deserve further investigation. In this context, H4R gene alterations (e.g., mutation, deletions, and amplifications) in different cancer types should be studied.

According to TCGA database, the gene expression profiles are obtained from primary solid tumors. Thus, we have to consider the cellular source of H4R expression in tumors. They comprise epithelial tumor cells and cells of the tumor microenvironment including infiltrating immune cells and endothelial cells that could also expressed H4R. Therefore, results of H4R gene expression should be confirmed by means of other methodologies including immunohistochemical studies in which not only the H4R protein expression but also the receptor-expressing cell type could be identified.

In this regard, numerous studies of the H4R protein expression and its localization have already been performed in different types of cancers including ESCC, GC, CRC, CHOL, breast cancer and melanoma (Medina et al., 2008; Fang et al., 2011; Meng et al., 2011; Zhang et al., 2012; Wang et al., 2014; Massari et al., 2017; He et al., 2018). Research data demonstrated the H4R positive immunoreactivity, especially in cancer epithelial cells. In most cases, differential expression of H4R between normal and malignant tissue assessed by immunohistochemistry was in accordance with the TCGA H4R gene expression.

It is important to point out that the specificity of the commercial H4R antibodies is a concern considering the non-specific binding effects. There are four different criteria to check the specificity of an antibody including the use of knockout cells, genetic knockdown of the expression in cells, cells recombinantly expressing closely related receptor subtypes, in which the reactivity of a specific antibody have to markedly decrease for genetic knockdown approach or be absent for the others (Seifert et al., 2013). Another criterium is the use of additional antibodies recognizing different epitopes but producing comparable results. In the latter, the presence of H4R splice variant isoforms, gene alterations and post-translational modifications should be considered in the analysis. Anyway, in most reported cases some efforts were made to check specificity of the H4R antibody, which include use of siRNA for H4R down-regulation, cells that do not expressed H4R and other methodologies such as Western blotting, FISH.

In addition, H4R expression was associated to prognosis in terms of OS in some cancer types, suggesting that H4R might represent a novel potential prognostic biomarker, which could complement routine histopathological analysis for some cancer types. Further immunohistochemical studies in a large number of patients’ tumor samples using antibodies with validated specificity should be dedicated to carefully defined the association between H4R expression and cancer prognosis.

Considering the potential different expression according to race, gender, and age, generalization of results should be avoided. Some of the studies investigating the H4R positive immunoreactive cancer cells and also the presence of polymorphisms of H4R were performed in Chinese population and therefore the level of H4R expression and the presence of H4R polymorphisms should be also corroborated in other ethnic groups.

A comprehensive study of H4R isoforms and their associated signaling pathways should be performed in different cancer types in order to evaluate whether a particular isoform is modulated by therapeutics or is involved in cancer progression. In this sense, pharmaceutical efforts could be focus in the development of drugs targeting specifically an isoform or selectively a signal transduction pathway.

H4R ligands exhibit a complex pharmacology, which is related to numerous factors including tissue variability in histamine-induced signaling pathways, functional selectivity, intra and interspecies differences in potency and selectivity, structural homology with H3R, splice variant isoforms, and polymorphisms that could preclude H4R function, together with impairment expression in pathological conditions. Therefore, a careful interpretation of H4R ligands’ effects *in vivo* should be performed.

Evidence from independent research groups support the introduction of highly potent and selective H4R agonists activating signal transduction pathways in tumor cell types that could contribute to advance in current tumor therapy paradigms in terms of a targeted therapy. However, although all preclinical studies that were performed using H4R agonists revealed a clear antitumor effect associated to reduced tumor growth and, in some cases, metastatic potential, they were carried out in immunodeficient hosts, in which the role of the immune system in the response to therapeutics could not be evaluated. One of the major impediments in the bench-to-bedside progress is the use of trustful mouse models that recapitulate the complexity of human cancer and immune population within the tumor microenvironment. Considering the notable involvement of H4R in immunomodulation, it is imperative to validate the therapeutic efficacy globally, considering the role of immune responses across all malignancies, which in turn will determine the clinical outcomes. In this sense, a recent study in H4R KO mice show for the first time the key role of this receptor in the antitumor immunity in a model of triple-negative breast cancer (Sterle et al., 2019). Therefore, before moving forward to clinical trials, pharmacological relevance of specific H4R agonists must be corroborated in immunocompetent tumor-bearing animal models.

Considering differences in gene expression levels among age, gender, ethnic origin, which should also be evaluated at the protein level, H4R ligands with potential use in clinical settings need to be evaluated for safety and efficacy in different ethnic groups, men and women, and children and in various oncological diseases.

It is worth noting that histamine treatment demonstrated favorable outcomes in different malignancies. In this regard, histamine stands out as a promising drug for cancer treatment, as a single agent or even for its use in combination therapy, which deserves to be tested in clinical settings. The fact that histamine is approved to be used in humans reduces the gap between experimental work and the potential clinical application.

The challenge remains to use generated basic research, identifying novel molecular targets, for the development of personalized cancer treatment.

## Data Availability

The datasets analyzed for this study can be found in the TCGA, https://www.cancer.gov/about-nci/organization/ccg/research/structural-genomics/tcga.

## Author Contributions

MN, KF, and VM wrote the manuscript. VM supervised the work.

## Conflict of Interest Statement

The authors declare that the research was conducted in the absence of any commercial or financial relationships that could be construed as a potential conflict of interest.
