# Supplementary figures and images for: Pathophysiological Role of Histamine H4 Receptor in Cancer: Therapeutic Implications
Source: Front Pharmacol. 2019 Jun 5;10:556. doi: 10.3389/fphar.2019.00556 (PMC6560177; doi:10.3389/fphar.2019.00556)

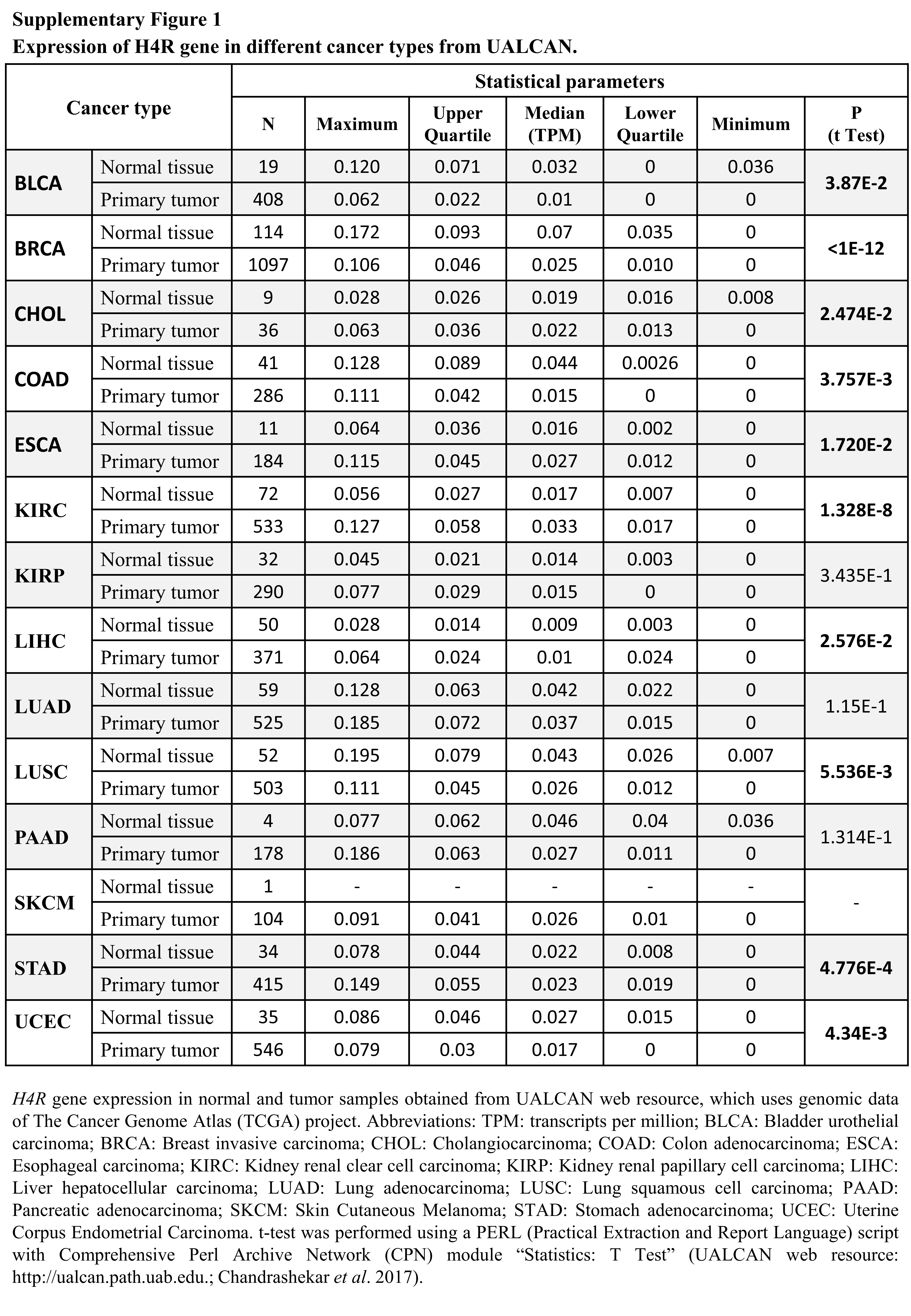

Supplement: Supplementary file 1 [file Image_1.TIF]

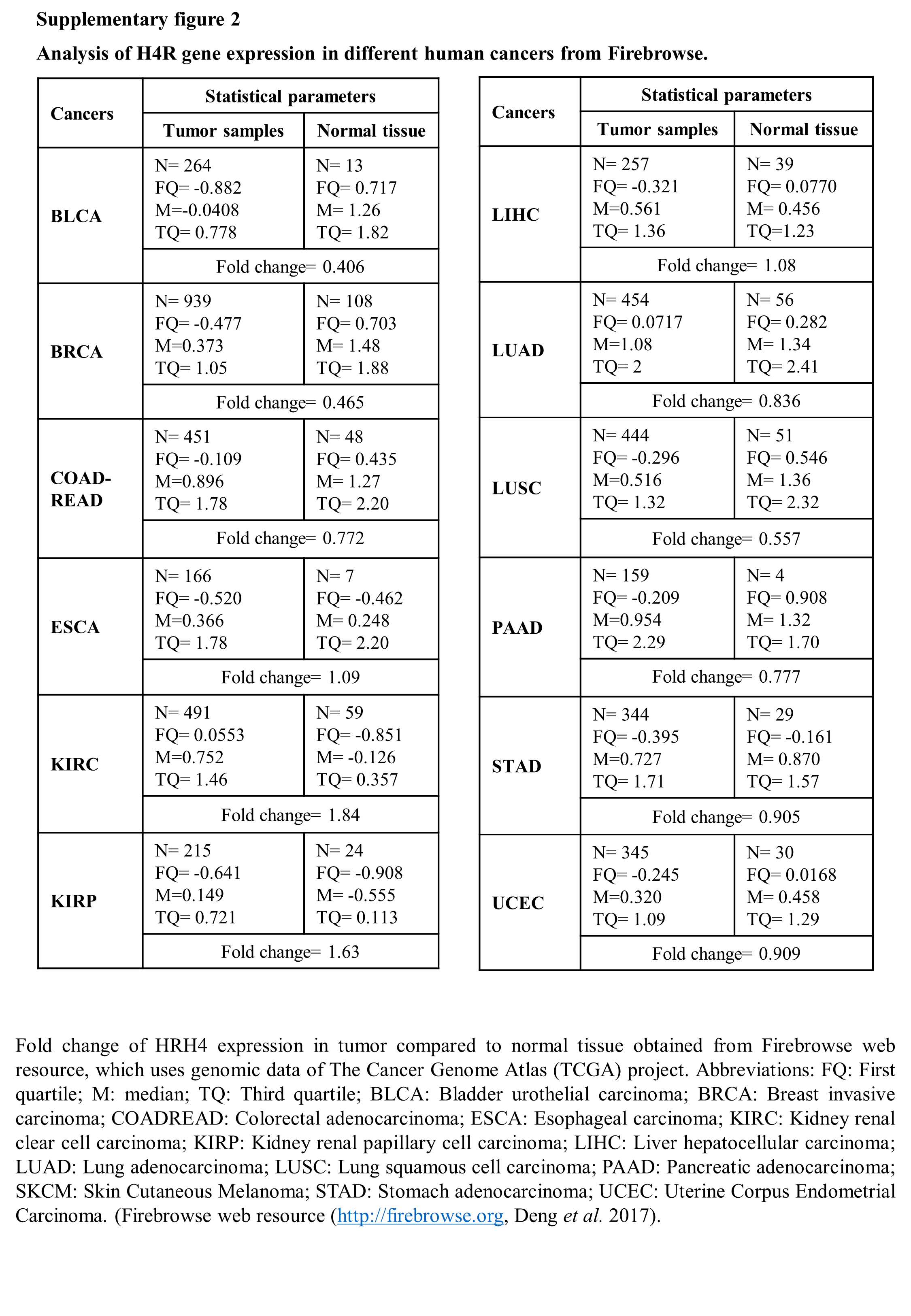

Supplement: Supplementary file 2 [file Image_2.tif]

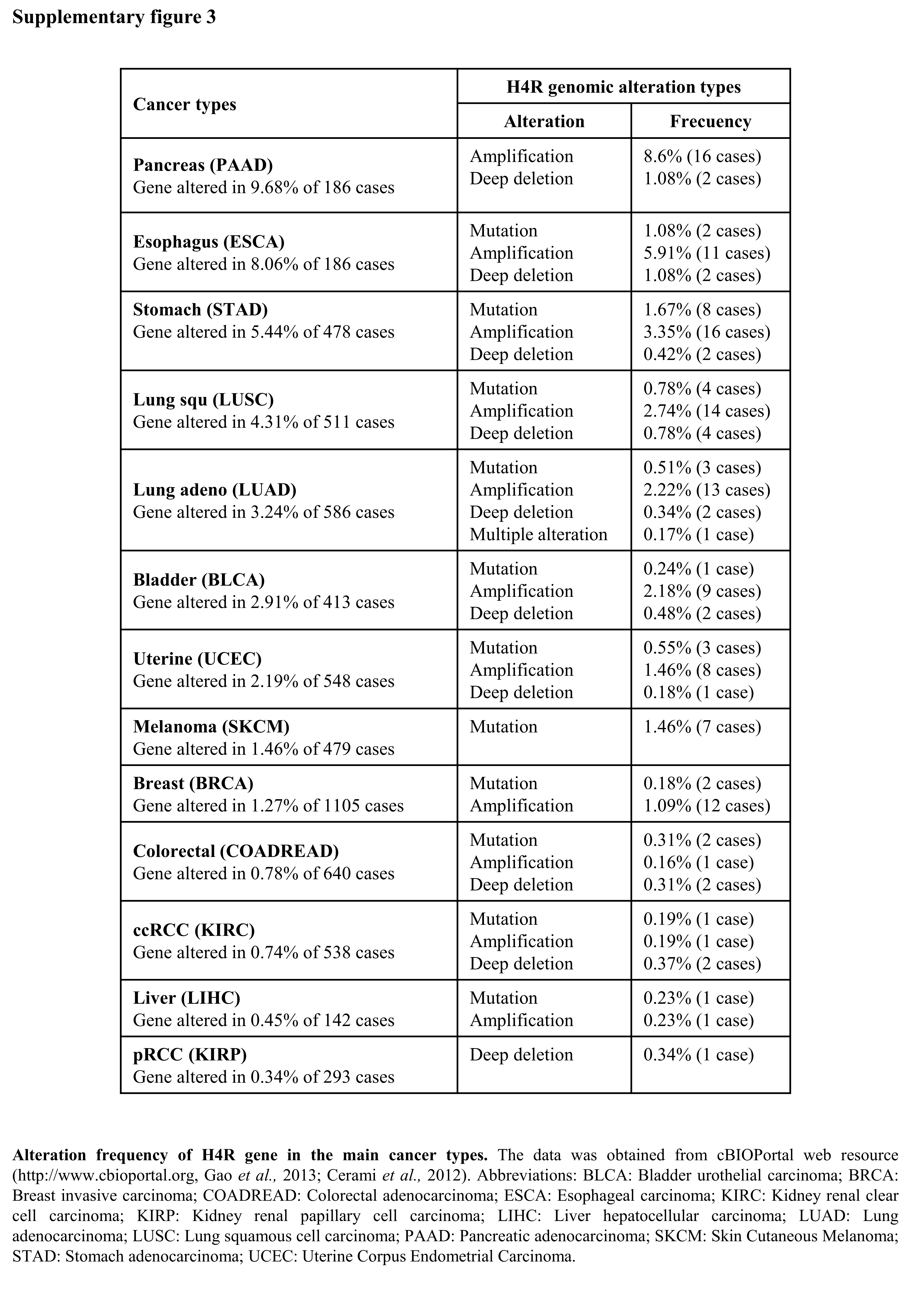

Supplement: Supplementary file 3 [file Image_3.TIF]
